# Supplementary material for: Chromothripsis during telomere crisis is independent of NHEJ, and consistent with a replicative origin
Source: Genome Res. 2019 May;29(5):737–49. doi: 10.1101/gr.240705.118 (PMC6499312; doi:10.1101/gr.240705.118)
Supplement: Supplemental Material [file supp_gr.240705.118_Supplemental_file_1.zip › contigs/annotated_contigs/DB76/contig.2.DB76_length_670_mean_cov_13.6358208955.docx]

**DB76_length_670_mean_cov_13.6358208955**

```
CTACCACACCTGGCTAATTTTTATATTTTTAGTAGAGATGAGGTTTCACCATGTTGGCCAGGCTGCTCTTGAACTCCTGACCTCAGGGG
 >chr5:163523428-163523775 - E=5e-194 p=1e-02
ATCCACCCGCCTCAGCCTTCCAAAGTGCTAGGATTACAGGTGTGAGCCACCGTGCCTGGTCTAGACATCTTTTATTCAGAAATTACATA

CATTGTCTCTAAAAACAATGGGCAGAAAAGTCAAACTAAGGAGCCTGAGACAAAAACAAAAGTATCATTTCATCACCTGCCAAACTCAC

AACCCTTCAGAAAGGGAGGAAGAAGAAGAAGAGATGAGCTATTTGCCAACTAAGAATATCTCATTAAAGCCACTTGAAGC|ACACACT|

TGTGTGTGTGTGTTCCAGTTGCTCTGGTTGTATAACAAGTTATCCTAAATATTAGTGCTTCAAACAATGATAAAATTTACACTTTACTT
>chr5:163517502-163517818 - E=2e-178
ACAAATCTGAAGTTTGGGCAAGACTTAGTGAGAACAGTTTGTTTCTGTTTTACTCGGCATCCCTTGGGGTGGCTGGAAGGGAGGAAGCT

GGAATCATCTGAAAGCTTATTCACTCACATTTCTGGTAGTTATCTGTAGACTGTGTTTAATTGGGGTCATTAGCAGAAACAAGTAAACA

CCGTGGCTTTTCCATGTGGCCTGGGGTTTCTTTCTCACAACATGATTGC
```
